# Vaccine Coverage in Children Younger Than 1 Year of Age during Periods of High Epidemiological Risk: Are We Preparing for New Outbreaks?

**DOI:** 10.3390/children9091334

**Published:** 2022-09-01

**Authors:** Valeria Herdea, Raluca Ghionaru, Claudiu N. Lungu, Eugene Leibovitz, Smaranda Diaconescu

**Affiliations:** 1 Doctoral School, “George Emil Palade’’ University of Medicine, Pharmacy, Science, and Technology of Târgu Mures, 38 Gheorghe Marinescu Str., 540139 Târgu Mures, Romania; 2 Romanian Association for Pediatric Education in Family Medicine, 021507 Bucharest, Romania; 3 Department of Surgery, Clinical Country Emergency Hospital Galati, 800578 Galati, Romania; 4 The Pediatric Infectious Disease Unit, Soroka University Medical Center, Beer-Sheva 85025, Israel; 5 Faculty of Health Sciences, Ben-Gurion University of the Negev, Beer-Sheva 84105, Israel; 6 Medico-Surgical Department, Titu Maiorescu University of Medicine and Pharmacy, 031593 Bucharest, Romania

**Keywords:** measles epidemic, COVID-19 pandemic, vaccine coverage, parental decision determinants

## Abstract

**Background**: According to WHO, infectious disease control can be achieved if the vaccine coverage (VC) exceeds 90%. In recent years there has been a declining trend in VC which could lead to the recurrence of infectious diseases. **Objectives**: The study analyzed the determinants of VC and of parental decisions regarding immunization in children aged 0–1 year monitored during two high-risk epidemiological periods (the measles epidemic and the COVID-19 pandemic period). **Methods**: A retrospective observational cohort study-data regarding vaccination of children younger than 1 year of age during the periods January 2019–June 2019 (measles epidemic) and January 2020–June 2020 (COVID-19 pandemic) were analyzed. 2.850 children from 2019 and 2.823 children from 2020 were enrolled. Family physicians interacted with 2840 parents or legal guardians in 2019 and with 2800 parents or legal guardians in 2020, during the infants’ consults providing medical information and answer to their questions and worries regarding their immunization. Data on immunization schedules on the determinants of parents’ decisions regarding vaccination were evaluated. **Results**: During 2019–2020, VC has followed a declining trend for each type of vaccine included in the Romanian National Immunization Program; the most affected were infants aged 9–12 months during both periods: in 9-month aged infants, the MMR vaccine VC was 67.49% in 2019 vs. 59.04% in 2020 (*p* < 0.004). In the 12 months aged infants, the MMR VC was 64.29% in 2019 vs. 55.88% in 2020 (*p* < 0.005). For the Hexavalent vaccine administered at the age of 11 months, the VC was 71.59% in 2019 vs. 62.08% in 2020 (*p* < 0.001). The determinants of parents’ decisions regarding vaccination included parental hesitance 2019—25% vs. 2020—35%, fear on side effects 2019—32% vs. 2020—45%, vaccination denial 2019—7% vs. 2020—10%. Conclusion: We found a declining trend in the VC in Romania during the epidemic and pandemic periods. The decrease in VC for MMR generated a major risk for new measles outbreaks Permanent awareness educational campaigns regarding infectious disease risk are needed, accompanied by the empowerment of primary care and the emergence of an immunization management program based on national regulatory legislation.

## 1. Introduction

Vaccine coverage (VC) is the key factor in estimating the performance of immunization systems. According to Worlds Health Organization (WHO) experts, achieving an optimal VC of over 90% for a vaccine designed to prevent an infectious disease allows the control of that disease. Maintaining the VC consistently over 95% globally for a vaccine allows the eradication of targeted diseases. However, during the COVID-19 pandemic, in the spring of 2020, 22% of infants in the WHO European Region had their vaccination courses interrupted [1,2,3].

In 1796, Edward Jenner applied the first smallpox immunization procedure but only 160 years later, in 1958, the WHO launched a worldwide campaign to eradicate smallpox. Barely in 1977, almost 200 years distance from Dr. Jenner’s first attempt, smallpox could be considered eradicated from the entire globe [4]. Regarding the impact on global population health status, Plotkin and Mortimer (1988) established the impact of vaccination as follows: with the exception of safe water, no other modality has had such a major effect on mortality reduction and population growth” [5].

Achieving a level of protective VC requires sustained activity by the medical staff, from expanding the communication between the medical professionals and the public and the recognition of the need to permanently raise the level of awareness for the infectious diseases vital risk among patients, parents, or relatives in the case of minor children, up to the activity of vaccinating the population. The digital age has allowed the general population to get access to all kind of information. Access to medical information has led to an improvement in VC in the general population but has also facilitated the emergence of parents who refuse vaccination, as well as of a category of parents defined as” hesitant” who avoid/postpone vaccination [6].

The access to the correct information provided by medical sources, may lead informed parents to take an easily and early optimal decision for their children, finally contributing considerably to the protection of their children from the danger of infectious diseases through vaccination [7,8].

In Romania, the National Immunization Program (NIP) includes 11 vaccines, of which by the age of 12 months, the infant will receive 10 vaccines complex products (3 hexavalent, 1 trivalent, and 5 monovalent): at birth, in maternity, prevention of severe forms of tuberculosis (BCG) and hepatitis B (AHB); at 2, 4, 11 months of age- Hexavalent vaccine (6 strains: Hepatitis B, Haemophilus influenzae type B, Diphteria- Tetanus- Pertussis- Poliomyelitis IPV)+ pneumococcal vaccine (Prevenar 13); at 9 months–12 months of age: measles-mumps-rubella). Due to the decrease in the incidence of measles infection in Romania, from October 2020 the early vaccine administration at the age of 9 months was interrupted leaving the administration of the first dose at age of 12 months with a booster at the age of 5 years [9].

VC, as a medical and social phenomenon, encounters political, social, cultural, traditional, language, educational level and legislation obstacles. In addition, access to the vaccine product, its acceptance and the emergence of high-risk epidemiological situations (like the measles epidemic or COVID-19 pandemic) may complicate the VC in many area of the world [10].

The goal of this study was to analyze the determinants of VC in children aged 0–1 year in Romania, by monitoring two high-risk epidemiological periods: the measles epidemic and the COVID-19 pandemic period.

## 2. Materials and Methods

At the level of primary care, this observational, retrospective, cohort study was conducted on the infant population aged 0–12 months living in several Romanian cities (Bucharest, Cluj-Napoca, Bistrita, Iasi, Braila, Sibiu, Pantelimon-Ilfov) during the period 2019–2021. In Romania, the main vaccination provider is the family physician. The study was performed by the members of the-“Vaccine Advocacy” team of the Romanian Association for Pediatric Education in Family Medicine”. The “Vaccine Advocacy” group developed during the last years a large educational program named” My Family School”, dedicated to family’s health education. As part of this major program, an additional program, the “Protect Life Ro-Program” providing education for the preventive life-style promotion, was developed targeting the age group of 0–1 years and educating future and young mothers.

The data collected respect the principle of confidentiality, without the possibility to identify the persons whose data have been collected. Each participating physician signed the agreement to enter the study and use the data transmitted for research purposes only. The informed parental agreement regarding the act of vaccination was a monitoring criterion recorded in the child’s electronic file. The study complied with the Romanian legislation (Law 190/2018) and GDPR—The General Data Protection Regulation 679/2016. The study was approved by the hospital’s Helsinki ethics committee.

The data were obtained from the archive computerized files of 12 family medicine offices in several major Romanian cities (Bucharest, Cluj-Napoca, Bistrita, Iasi, Braila, Sibiu, Pantelimon-Ilfov). The VC data were summarized for the first 6 months of 2019 and for the first 6 months of 2020. The mothers enrolled went through an educational process during their pregnancy, completed in the family medicine office by the medical staff (physicians and nurses). Data was confirmed/completed from the medical office documents registered in the National Electronic Register of Vaccinations. The records of the vaccinated children were analyzed for each month. The records summarized infants’ data, by age, gender, and the way of informed refusal of vaccination (forms signed by parent or legal guardian) after prior parental counseling by the vaccinating medical staff.

Missing vaccination appointments were recorded for various reasons provided by parents or physicians, such as: “acute febrile illness” (as a temporary contraindication for vaccinating an infant), request for delaying vaccination from the parents for personal reasons, family working temporarily abroad/cross-border families, and lack of consent for vaccination, from both or one of the parents).

The study inclusion criteria were: 1. infants between 0–12 months of age; 2. follow-up by the same family doctor for at least 12 months; 3. complete medical history available; 4. presence of one parent or legal guardian in the medical office during child evaluation and vaccination.

The study exclusion criteria were: 1. children older than 1 year; 2. follow-up completed by several family doctors until the age of 12 months; 3. incomplete medical data; 4. patients where a catch-up schedule was needed after 12 months of age.

Data regarding vaccination of children younger than 1 year of age during the periods January 2019–June 2019 (measles epidemic) and January 2020–June 2020 (COVID-19 pandemic) were analyzed. Family physicians interacted with 2840 parents or legal guardians in 2019 and with 2800 parents or legal guardians in 2020, during the infants’ consults, providing medical information and answering to their questions and worries regarding the child immunization. Parents were counseled by medical teams, about the risk and severity of the infectious diseases discussed and the benefits of vaccination. Data on immunization schedule, parental attitudes regarding vaccination (hesitant parents, vaccination denial parents) and on the determinants of parents’ decisions regarding vaccination acceptance for their children (from fear regarding side effects of vaccines to fake news/misinformation/disinformation influence on parents’ decision or “pro-disease” online campaigns followers (against-vaccination online campaigns) were recorded during the study.

In addition, the study followed a special category of children, belonging to cross-border families These children’s parents worked abroad temporarily. The data considered two circumstances: children who were under relatives care in Romania for the periods when the parents worked abroad and children who moved with their families from a country to another country during the periods when the parents worked temporary in European countries. For this group, data were obtained from relatives who joined the children for vaccination appointments or from parents who were present in the family physicians’ offices in the vaccination day.

For data regarding the total number of newborn babies from Romania, for the years 2019 and 2020 we used statistics of the Romanian National Institute of Statistics. In respect to data regarding vaccine supplies issues during the state of pandemic emergency declared in Romania between 16 March–15 May 2020, we used data from National Center for Study and Control for Infectious Diseases, comparing the years 2019–2020 and the months of March-April-May, according to the period when we registered the lowest vaccine coverage values.

## 3. Results

**Figure 1:**
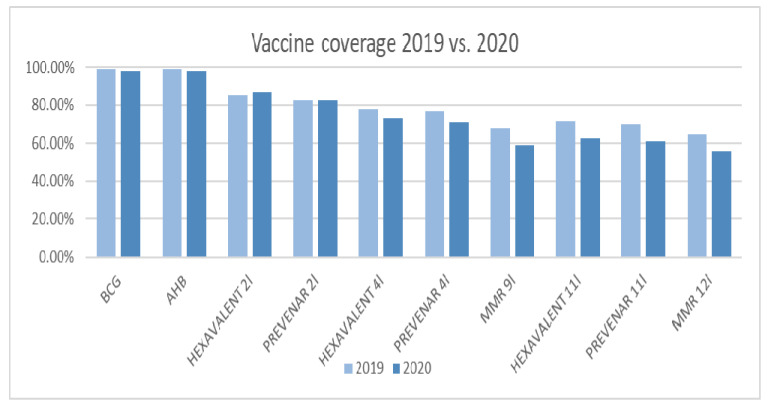
Vaccine coverage 2019 vs. 2020. VC was analyzed for each type of vaccine included in The National Immunization 0–12 months of age group—Vaccines included in The National Immunization Program: Calmette Guerin (tuberculosis), AHB—Hepatitis B, MMR—mumps-measles-rubella, Prevenar 13, Hexavalent—(Hepatitis B, Haemophilus influenzae type B, Diphteria-Tetanus-Pertussis-IPV Poliomyelitis).

A total number of 5673 infants aged 0–12 months were included in the study. The yearly distribution of the two groups of infants was 2850 infants (born 2018–2019) during 2019 and 2823 (born 2019–2020) during 2020 (Figure 1).

In the same study group were included children whose parents worked temporary abroad. They number raised from 120 children in 2019, (2.11% of all children from the study group) to 250 children in 2020 (4.40% of all children from the study group) (Table 1).

According to National Institute of Statistics, in 2019 were registered 203,109 newborn babies and in 2020—178,609 newborn babies. The study group of 2019—2850 infants-represent 1.40% off all newborn babies from Romania in 2019. The study group of 2020—2823 infants represent 1.58% off all newborn babies from Romania in 2020 (Table 2).

Vaccine coverage was analyzed for each type of vaccine included in National Immunization Program (Table 3).

Comparison between 2019–2020 were made for the following vaccines: BCG—Bacillus Calmette Guerin (tuberculosis), AHB—Hepatitis B, MMR—mumps-measles-rubella, anti-pneumococcal vaccine (Prevenar 13), Hexavalent vaccine (Hepatitis B, Haemophilus influenzae type B, Diphteria-Tetanus-Pertussis-Poliomyelitis IPV)+ anti-pneumococcal vaccine (Prevenar 13).

A significant decrease in VC was recorded from 2019 to 2020 in respect to pneumococcal vaccination at 4 months of age, MMR at 9 months of age, Hexavalent vaccination and pneumococcal vaccination at 11 months of age, and MMR at 12 months of age (Figure 1 and Table 3).

Regarding the pneumococcal vaccination at 4 months of age, a 5.62% decrease in VC was recorded between 2019-76.52% to 70.90% in 2020 (*p* < 0.04%). For MMR at 9 months of age, a VC decline of 8.45% was recorded from 67.49% in 2019 to 59.04% in 2020 (*p* < 0.004).

At 11 months of age, the Hexavalent vaccine VC has dropped by 9.51% from 71.59% in 2019 to 62.08% in 2020 (*p* < 0.001), the lowest values being recorded during March-April-May 2020. The data from National Center for Study and Control for Infectious Diseases for the years 2019–2020, for the months of March-April-May, has reported for 2020 vaccines supplies problems. The official report regarding Hexavalent vaccine supplies shortage during the state of pandemic emergency declared in Romania between 16 March–15 May 2020 is presented in Table 4.

At the age of 11 months, the pneumococcal vaccine coverage VC also dropped by 8.62% from 69.70% in 2019 to 61.08% in 2020 (*p* < 0.05).

The VC for MMR at 12 months of age reached 64.29% in 2019 and 55.88% in 2020, a drop of 8.4% (*p* < 0.005) (Figure 1, Table 3 and Table 5). The Official Report regarding MMR vaccine supplies shortage during the state of pandemic emergency declared in Romania between 16 March–15 May 2020 is presented in Table 5.

No differences between 2019 and 2020 were recorded in the VC in the immunization of infants younger than 4 months.

When the parental attitude regarding children immunization was analyzed, data recorded in the family physicians’ offices showed an increasing percentage of hesitant parents from 25% (among all parents who completed the questionnaires) in 2019 to 35% in 2020, denial of vaccination raised from 7% in 2019 to 10% in 2020 and fear regarding side effects of vaccination from 32% in 2019 to 45% in 2020 (Table 6).

Regarding the determinants of parental decision regarding children immunization, for 2019, during the measles epidemic, 1 of 4 parents asked the physician (during the child examination before vaccination) at least 1 question, whose source was not in accordance with the scientific truth, based on fake news accessed from the Media (TV/online press/blogs. In 2020, during the COVID pandemic, 2 of 4 parents ask the physician before vaccination at least 1 question whose source was not in accordance with the scientific truth, based on fake news accessed from the Media (TV/online press/blogs. From the categories of vaccination denial parents, 198 were recorded as online pro-disease campaigns followers (against-vaccination online campaigns supporters’’) in 2019 and 280 in 2020.

## 4. Discussion

The main findings reported in this study showed that the VC decrease for many routine childhood vaccines was more severe in 2020 compared with 2019, emphasizing that the pandemic may have affected the parental decisions regarding child immunization. In addition, the parents were found to be influenced by media and by fake news, misinformation and disinformation and their attitudes on vaccination might have been a major determinant in the VC decrease reported [11].

The vaccination is free and voluntary, but a clear regulatory legislation is missing in Romania. During the measles epidemic in 2019 and in the pandemic period in 2020, over the same months of March-April-May, many children belonging to the age groups of 9, 11 and 12 months were ill with the disease and were not vaccinated according to the routine immunization schedule, being rescheduled for later appointments. The missing of a scheduled immunization visit to doctor’s office, (with the usual reasons as “illnesses” or “silent absence attitude”), may frequently point for a hesitant parent. On the other hand, based on the number of children born in Romania every year [12], the Ministry of Health and the Public Health Directorates act by preparing the yearly vaccine supplies at the country level. However, the VC was affected by problematic management of the vaccinal resources, when the management of vaccines delivery to family physicians’ offices was a real problem for the Public Health Directorates, as a result of the magnitude of the pandemic, which affected also the medical personnel involved in the pandemic surveillance. For 2020, a shortage in the supply of vaccines was recorded in comparison with 2019-data corresponding to the period when the pandemic state of emergency was established in Romania [13].

The Hexavalent vaccine VC has dropped by 9.51% from 71.59% in 2019 to 62,08% in 2020, the lowest values being recorded during the months March-April-May 2020, period of the state of pandemic emergency (declared in Romania between 16 March–15 May 2020). Similarly, the lowest percentage of VC (55.8%) was recorded for the MMR vaccine administered in 12-month infants during April 2020. During both years studied, the age groups of 9, 11 and 12 months had a lower VC compared with younger ages while in the first 2–4 months of the life the parents were more compliant to physician’s recommendations.

During 2019–2020, VC has followed globally a declining trend. If in the 2017–2019 period, measles epidemic affected the US and many European areas only [14,15,16], the COVID-19 pandemic 2020 until 2022 affected the entire World [17,18,19]. The decrease of VC remains a global phenomenon, a process induced on one hand, by the major epidemiological risk (epidemic, pandemic) and on the other hand by “infodemia” manifested frequently in media. Infodemia, defined as “an overabundance of information”, makes difficult for people to find trustworthy sources and reliable guidance when they need it, and is represented by” mixed rumors, stigma, and conspiracy theories during major public health emergencies” [20,21,22].

Between 2017–2020, Romania, like many other countries, faced the vaccine-related fake-news infodemic phenomenon, but was also affected by the need to take care of a high percentage of cross-border citizens (working outside the Romanian borders) without access to adequate medical services, having the severe VC declining trend as a final result [23,24,25,26,27,28,29].

Of course, VC is a phenomenon dependent on the population’s access to country’s medical services. Globally, the statistics of the WHO and the World Bank showed for 2015–2016 over 400 million people worldwide without access to basic medical services. In 37 low- and middle-income countries, 6% of population was found to be pushed to extreme poverty (income of $1.25/day) because the need to pay for health services [25].

In the United States where health services represented an average of 14% of the gross domestic product, 28 million people are without health insurance [26].

In Romania, with a population of 19,184,334 people, according to Health Ministry, 4.8 million people were reported in 2022 without health insurance and 2.52% of the Romanian population doesn’t have access to a family physician in their living area [28,29,30,31,32].

In the epidemic context 2018–2019 or pandemic period 2020–2022, VC has experienced global oscillations. The risk of disease increases with the recurrence of an infectious disease previously considered controlled (by permanently applied vaccine exercise) or eradicated if the VC has constantly reached 90% in a population. On the other hand, the phenomenon of hesitation to vaccinate has occurred and increased significantly in an epidemic or pandemic context. This is generated by the promotion of the exacerbated perception of potential risks related to the act of vaccination versus the real risks of the disease and the consequences induced by infections on the quality of human life or the benefit of vaccination, which are facts scientifically proven [32,33].

Considered eradicated in the US in 2002, measles has returned through an epidemic triggered in 2018–2019 in a religious minority cluster, with rapid expansion in several US states. The number of cases reached 1282 in a few months [34]. By comparison, between 2017–2019, the measles epidemic in Romania registered 17.133 cases of them 3665 under the age of 1 year. There were 74 deaths due to measles reported in the European Unity, of which 64 deaths in Romania alone [35,36].

The recent pandemic of coronavirus-2019 (COVID-19) has had an unprecedent impact on adults severely affected by COVID-19 infection. The number of children hospitalised for COVID-19 had immediately appeared lower than adults. It has been assessed that children and young people aged under 20, often asymptomatic, have a susceptibility to the infection which is about the half that of people aged more than 20. Pregnant women who are positive to SARS-CoV-2 are generally asymptomatic in a higher percentage than the general female population infected by the same virus, probably because they are generally younger. Despite there being a low possibility of vertical transmission, a baby whose mother is positive to COVID-19 may have adverse responses, such as foetal distress, preterm birth, respiratory difficulty and death [37,38].

Regarding the year 2020, the pandemic induced by the SARS-CoV-2 virus led in Romania to 479,634 confirmed cases, of which 11,530 people died by 01 December 2020 [39]. Globally, at the same time, there were 62.7 million confirmed COVID-19 cases and 1,460,477 deaths due to severe forms of SARS-CoV-2 virus infection [40]. July 2022 statistics for Romania show 3,063,647 COVID-19 cases and 66,000 deaths [41]. In the meantime, global statistics show 581,943,861COVID-19 cases and 6,419,449 deaths [18].

The pandemic has led to a significant reduction in VC globally, especially in children under 12 months of age. It is estimated that during COVID-19 VC of measles dropped below 80%, including the US, the main reasons being considered to be the isolation at home of the population during the state of pandemic emergency and the increasing phenomenon of parental hesitation for vaccination due to “infodemia” [11,16,21].

In Romania, the effects of the pandemic during 2020 materialized through a reduction by 20–30% fewer infants attending the family physicians’ offices, particularly during the March-May emergency period, compared with the same period of the previous year. As shown in our study, compared with 2019, when the “temporary contraindications to vaccination” during March-May referred to infants with “acute febrile diseases”, in 2020 for the same period, the percentage of hesitant parents who postponed the vaccination of infants increased considerably. In the same period, VC for MMR vaccines dropped under 60%, thus creating the major problem leading to measles recurrence.

During the state of emergency declared in Romania between 16 March–15 May 2020, the supply flow with many vaccine products was discontinuous. As a result, VC, especially for the 11-months age group dropped below 70% for Hexavalent vaccine [41]. However, VC for 2 months and 4 months-aged infants remained relatively constant compared to the previous year, as efforts were made by primary care medical staff to communicate with parents and mobilize them to vaccinate these infants.

About 75% of parents were found to trust the family physicians and the other medical staff for their advice in regard to information related to the vaccination phenomenon and its acceptance [41], but a significant percentage use the internet or read news from media. Recent research showed that only 5% of vaccination information sources accessed by parents on Google or other social media networks are in accordance with the scientific truth and 35–87% of the sources accessed by parents offer low-quality information, with the possible development of a hesitant behavior of postponing the vaccination [36,37].

In Romania, vaccination is free and not mandatory, according to the National Vaccination Program. Parental education during pregnancy is not compulsory. Consequently, the information about vaccines that the parent acquires does not always have a valid, medical and scientific source. Non-compliant sources may also inform parents and may lead to wrong decisions about infant health and also to postponement or refusal of vaccination. Research conducted in Romania in 2019 during the measles outbreak showed that about 30.3% of parents adopted a strategy of postponement, 27.7% declared vaccine hesitancy and 11.7% refused to vaccinate their children [18]. The vaccine hesitancy phenomenon was showed to increase globally between 2018–2019; in 2018, in Europe, Bulgaria had the lowest reported confidence in the safety of vaccines in the EU, with only approximately 66% of respondents believing vaccines are safe [41]; on the North American continent, in the same year, 19% of the Canadian population considered itself to be vaccine hesitant [42]; the World Health Organization declared vaccine hesitancy a “top 10 health threat” for 2019 [43].

Globally, the SARS-CoV-2 pandemic amplified during 2020 the phenomenon of parental vaccine hesitancy, putting pressure on primary care physicians to find new ways to communicate, interact and educate parents to understand the meaning of the prevention of the infectious diseases [44,45,46,47,48,49].

A group of special interest addressed in our study was represented by children belonging to“ cross-border families”. Many Romanian parents work abroad, often without a legal job; children are raised at poverty limit, without adequate access to healthcare services. They do not receive the preventive medical care in the country where their parents work and also lose the medical care in their mother country, remaining unvaccinated, and therefore being exposed to the risk of many infectious disease. In 2019, almost 18 million children in the EU (22.2% of the pediatric population) lived in households at risk of poverty or social exclusion [50]. Many times, Romanian children remain at home under the first-degree or second-degree elderly relatives care, being exposed to medical and social exclusion. The lack of a real family for these children is a challenge for community and the medical care for these children is a challenging process also for the family physician team [51].

There are some limitations related to this study, such as its short time period covered (first 6 months of 2019 vs. first 6 months of 2020) and, inherently, the lack of a bigger cohort study. In addition, many children probably were not tested for COVID-19 during the study period and therefore were not diagnosed with this condition. The presented data were extracted from many sources, some of them not entirely exact and reliable and some data was missing. Of course, the data presented here are representative for a specific Romanian geographic area and not for the whole Romania. Future studies are needed to monitor parental behavior during major epidemiological risk periods or regular periods.

In conclusion, we reported a lower vaccination coverage during two years of epidemic (measles) and pandemic (COVID-19) diseases, which may possibly lay the foundations for the recurrence of severe infectious diseases (like measles) or may affect the success of the worldwide preventive and therapeutic campaign against COVID-19. Fear regarding vaccines side effects, anti-vaccination media campaigns and the infodemic phenomenon play an important role in modeling parents’ decision to vaccinate their children. The management of the immunization process must be based on national clear regulatory legislation. Permanent awareness educational campaign regarding infectious disease risk and benefits of the vaccines are needed, starting with pregnant women and young mothers and finishing with senior citizens. The main vaccinator in Romania remains the family physician, therefore empowering the primary patient care at this level could be a solution for raising the VC and providing large scale protection of population general health.

## Figures and Tables

**Table 1:** Study group description.

1. Year	2019	2020
2. No. children	2850	2823
3. No. parents/legal guardians	2840	2800
4. Cross-border families children	120	250

**Table 2:** Study group demographic characteristics.

Year	No of Children Born in Romania/Year	No of Infants-Study Group	Percentage of Study Group Infants from all Children Born in Romania/Year
2019	203,109	2850	1.40%
2020	178,609	2823	1.58%

**Table 3:** Vaccine coverage: comparison between 2019 and 2020.

Vaccine	2019	2020	*p*-Value
BCG	99.18%	97.74%	0.115
Hepatitis B	99.18%	98.02%	0.184
HEXAVALENT 2 m	85.23%	86.85%	0.488
PREVENAR 2 m	82.55%	82.77%	0.932
HEXAVALENT 4 m	77.94%	72.95%	0.0695
PNEUMOCOCCAL 4 m	76.52%	70.90%	0.045554044
MMR 9 m	67.49%	59.04%	0.005
HEXAVALENT 11	71.59%	62.08%	0.001
PNEUMOCOCCAL 11 m	69.70%	61.08%	0.004
MMR 12 m	64.29%	55.88%	0.005

**Table 4:** Hexavalent vaccine supply in 2019 and 2020 ((Vaccine stocks according to National Center for Study and Control for Infectious Diseases accessed at: http://cnscbt.ro/index.php/situatia-stocurilor-de-vaccinuri [Romanian] (Accessed on 31 July 2022).

Reported Month	2019	2020
March	227.703	211.055
April	384.591	176.364
May	359.124	150.003

**Table 5:** Measles-Mumps-Rubella (MMR) vaccines supplies at the end of the months March-April-May 2019–2020 (Vaccine stocks according National Center for Study and Control for Infectious Diseases accessed at: http://cnscbt.ro/index.php/situatia-stocurilor-de-vaccinuri [Romanian] (accessed on 31 July 2022).

Reported Month	2019	2020
March	187.116	173.748
April	229.273	144.203
May	196.410	132.300

**Table 6:** Parental attitudes regarding vaccination.

1. Year	2019	2020
2. Hesitant parents	25% (710)	35% (980)
3. Vaccination denial parents	7% (198)	10% (280)
4. Fear regarding the side effects of vaccines	32% (908)	45% (1260)

## Data Availability

The datasets used and/or analyzed during the current study are available from the corresponding author upon reasonable request.
